# Artificial Intelligence Analysis of Ulcerative Colitis Using an Autoimmune Discovery Transcriptomic Panel

**DOI:** 10.3390/healthcare10081476

**Published:** 2022-08-05

**Authors:** Joaquim Carreras

**Affiliations:** Department of Pathology, Faculty of Medicine, Tokai University School of Medicine, 143 Shimokasuya, Isehara 259-1193, Japan; joaquim.carreras@tokai-u.jp; Tel.: +81-463-93-1121

**Keywords:** ulcerative colitis, artificial intelligence, machine learning, artificial neural networks, autoimmunity, transcriptome, immune checkpoint, immuno-oncology, *PD-L1*, immune microenvironment

## Abstract

Ulcerative colitis is a bowel disease of unknown cause. This research is a proof-of-concept exercise focused on determining whether it is possible to identify the genes associated with ulcerative colitis using artificial intelligence. Several machine learning and artificial neural networks analyze using an autoimmune discovery transcriptomic panel of 755 genes to predict and model ulcerative colitis versus healthy donors. The dataset GSE38713 of 43 cases from the Hospital Clinic of Barcelona was selected, and 16 models were used, including C5, logistic regression, Bayesian network, discriminant analysis, KNN algorithm, LSVM, random trees, SVM, Tree-AS, XGBoost linear, XGBoost tree, CHAID, Quest, C&R tree, random forest, and neural network. Conventional analysis, including volcano plot and gene set enrichment analysis (GSEA), were also performed. As a result, ulcerative colitis was successfully predicted with several machine learning techniques and artificial neural networks (multilayer perceptron), with an overall accuracy of 95–100%, and relevant pathogenic genes were highlighted. One of them, programmed cell death 1 ligand 1 (*PD-L1*, *CD274*, PDCD1LG1, B7-H1) was validated in a series from the Tokai University Hospital by immunohistochemistry. In conclusion, artificial intelligence analysis of transcriptomic data of ulcerative colitis is a feasible analytical strategy.

## 1. Introduction

Ulcerative colitis is a disease of the colon that is characterized by recurrent episodes of inflammation of the mucosa. It usually involves the rectum, and it can extend beyond toward the proximal areas of the colon continuously.

The onset of ulcerative colitis is usually gradual, and symptoms are progressive during several weeks. The patients usually present with diarrhea, sometimes with blood, abdominal pain, urgency, or tenesmus [1,2]. Systemic symptoms may also be present, including fever, fatigue, and weight loss [2].

Disease severity assessment is important for clinical management and includes the Montreal classification (mild, moderate, and severe) [3], and the Mayo scoring system that evaluates the stool pattern, most severe rectal bleeding of the day, endoscopic findings, and global assessment [4]. The diagnosis is based on the presence of chronic diarrhea of more than 4 weeks and the demonstration of active inflammation on endoscopy and chronic changes on the biopsy [2].

There are a series of features suggestive of ulcerative colitis on the biopsy, including crypt abscesses, crypt branching, shortening and disarray, and crypt atrophy. The epithelial layer is also affected and shows mucin depletion and Paneth cell metaplasia. The mucosa is inflamed, and increased lamina propria cellularity is found, along with basal plasmacytosis, basal lymphoid aggregates, and lamina propria eosinophils [2]. The histological features can be evaluated using the Geboes Score, the simplified Geboes Score [5], and others, such as the Robarts histopathology index and Nancy index [6].

We have recently described some of the immune microenvironment elements of the mucosa of ulcerative colitis patients and found that T lymphocytes and macrophages were important components of the inflammatory infiltrate [7]. The aim of this study was to use artificial intelligence analysis, using gene expression data, to identify the genes associated with the development of ulcerative colitis. This research is a proof-of-concept analysis using publicly available transcriptomic data to demonstrate that machine learning and artificial neural networks are useful for diagnosing ulcerative colitis and for understanding the pathogenesis.

## 2. Materials and Methods

A publicly available gene expression dataset of ulcerative colitis was searched at the National Library of Medicine, National Center for Biotechnology Information, webpage: https://www.ncbi.nlm.nih.gov/ (accessed on 5 July 2022). The dataset GSE38713 was selected [8].

The inclusion criteria for ulcerative colitis patients were the following: age between 18 and 65, and diagnosis of UC established at least 6 months before inclusion and exclusion of concomitant infection [8].

Active disease was defined using an endoscopic and histological scores. The Mayo sub score ≥ 2, and MATTS ≥ 3, respectively. The definition of inactive disease was based on endoscopic and histologic scores of Mayo sub score = 0 and MATTS ≤ 2, respectively; and a remission state for a minimum of 5 months before biopsy collection and remained inactive for at least 6 months after [8]. Uninvolved mucosa from patients with active ulcerative colitis was defined as a colonic segment with a completely normal endoscopic appearance, normal histology, and absence of any previous evidence of active disease [8].

The series comprised a total number of 43 biopsies, including 13 healthy controls, 8 inactive ulcerative colitis, 7 non-involved active ulcerative colitis, and 15 involved active ulcerative colitis [8].

This dataset contains gene expression data from a whole-genome transcriptional analysis of colonic biopsies from patients with histologically active and inactive UC, as well as non-inflammatory controls. Total RNA had been extracted by Rneasy Kit (Qiagen) according to the manufacturer’s instructions. The biotinylated cRNA was prepared according to the standard Affymetrix protocol. The sample hybridization protocol was the standard Affymetrix protocol. The sample scan protocol was the standard Affymetrix protocol using a Gene chip scanner 3000. Data processing: the data were analyzed with Bioconductor tools in R (http://www.r-project.org) (accessed on 3 August 2022) using GC-RMA as a normalization method. Next, a conservative probe-filtering step was performed, excluding those probe sets not reaching a log2 expression value of 5 in at least 1 sample. The sample platform identification was GPL570, [HG-U133_Plus_2] Affymetrix Human Genome U133 Plus 2.0 Array [8].

A basic tool to compare two or more groups of samples to identify genes that are differentially expressed across experimental conditions was initially used: the GEO2R tool, webpage: https://www.ncbi.nlm.nih.gov/geo/geo2r/ (accessed on 5 July 2022). The analysis options were the following: adjustment to the *p* values using the Benjamini & Hochberg (False discovery rate), auto-detect application of log transformation to the data, not the application of limma precision weights, and no forced normalization. For the analysis, the significance level cut-off was set at 0.05. The volcano and MA plot contrasts were control vs. active ulcerative colitis.

Several machine learning analyses, including artificial neural networks, were performed. For all statistical analyses, several software was used for data preparation, processing, analysis, and confirmation of results. The software included Microsoft excel 2016 (Microsoft Corporation), EditPad Lite (Just Great Software Co., Ltd., Phuket, Thailand), GSEA v4.2.3 (UC San Diego, Broad Institute, San Diego, CA, USA), JMP Pro 14 (JMP Statistical Discovery LLC, SAS, Cary, North Carolina, USA), Minitab 21 (Minitab, LLC, State College, PA, USA), IBM SPSS Statistics 26 and modeler 18 (IBM), and RapidMiner Studio 9 (RapidMiner). GEO2R ran on R 3.2.3, Biobase 2.30.0, GEOquery 2.40.0, and limma 3.26.8. All the analyses were performed as described in our recent publications [9,10,11,12,13,14,15,16,17,18]. A detailed description of the artificial neural networks is given in references [9,10,13,16]. GSEA is described in reference [17]. Immunohistochemical procedures in references [11,12,15]. Machine learning in references [14,17,18]. For this analysis, a desktop equipped with a 12 core processor AMD Ryzen 9 5900X, 16 GB of RAM, and a GPU Nvidia GeForce RTX 3060 Ti was used.

## 3. Results

Results summary:

- Conventional gene expression analysis using volcano plot differentiated the expression of active ulcerative colitis vs healthy donors broadly using all the genes of the array.
- Gene set enrichment analysis (GSEA) using an autoimmune discovery panel showed enrichment toward the ulcerative colitis phenotype, highlighting the most relevant genes in the leading edge.
- Several machine learning and artificial neural network analyses predicted ulcerative colitis against healthy donors using the autoimmune discovery gene expression panel.
- A high expression of programmed cell death 1 ligand 1 (*PD-L1*, *CD274*) in ulcerative colitis was validated in an independent series using immunohistochemistry analysis for histological identification of protein expression.

### 3.1. Conventional Analysis Using the GEO2R Software

A conventional gene expression analysis was performed using the GEO2R software, which compared the gene expression between 13 healthy controls and 15 involved active ulcerative colitis.

Based on the adjusted p values, the 10 most important gene probes were associated with active ulcerative colitis, including *SLC6A14* (219795_at), *REG1B* (205886_at), *REG1A* (209752_at), *LPCAT1* (201818_at), *DUOXA2* (230615_at), CD55 (201926_s_at), C4BPB (208209_a_at), and KCND3 (213832_at), and associated with healthy controls, including *HMGCS2* (240110_at) and DPP10-AS1 (236351_at).

**Figure 1:**
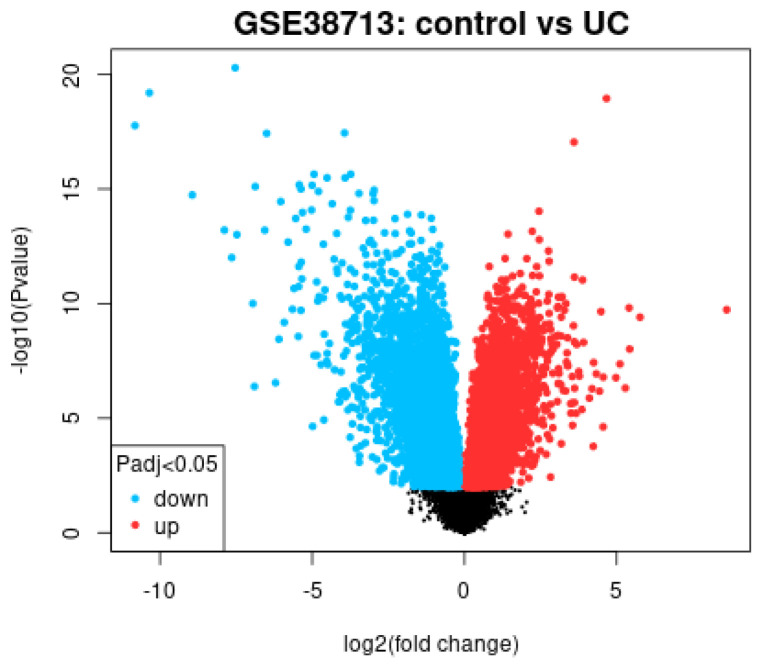
Volcano plot. This type of plot is useful to identifying genes that differ significantly between healthy controls and active ulcerative colitis. This type of graph relates fold change to *p* values. Upregulated genes are highlighted in red and downregulated in blue.

This type of analysis used all the genes of the array (Figure 1). Therefore, the results are of limited interest as they lacked pathway analyses. Nevertheless, the *CD274* (*PD-L1*) gene probe (227458_at) was identified, with an adjusted *p* value of 1.73 × 10^9^, and was associated with active ulcerative colitis.

### 3.2. Gene Set Enrichment Analysis (GSEA) Using an Autoimmune Discovery Panel

A panel of 755 genes was selected from the Affymetrix Human Genome U133 Plus 2.0 Array. For this analysis, if one gene had several probes, the probes were collapsed to the maximum expression so that each gene had only one expression value. This panel, named the autoimmune discovery panel, contained genes closely associated with germline variants across nine different autoimmune diseases or relevant to the immune response. The autoimmune disease coverage included multiple sclerosis, rheumatoid arthritis, systemic lupus erythematosus, type 1 diabetes, ankylosing spondylitis, celiac disease, inflammatory bowel disease (Crohn’s disease and ulcerative colitis), and psoriasis. The disease-associated genes were curated from studies available through ImmunoBase (www.immunobase.org; www.opentargets.org (accessed on 3 August 2022).

**Figure 2:**
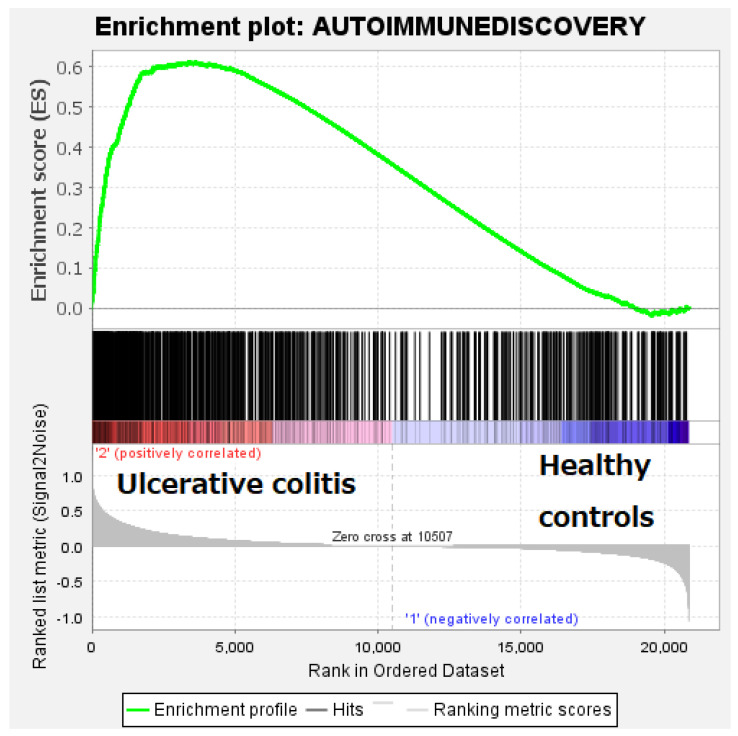
Gene set enrichment analysis (GSEA) using an autoimmune discovery panel. The GSEA analysis confirmed that a priori set of genes of the autoimmune discovery panel showed a significant difference between ulcerative colitis and healthy controls. The analysis showed enrichment toward ulcerative colitis. The most relevant genes of the leading edge were *IL1RN*, *MMP3*, *OSMR*, *FCGR3B*, *FCGR3A*, *TNC*, *TNFRSF6b*, *CD274* (*PD-L1*), *PLAU*, and *S100A9*.

Gene set enrichment analysis (GSEA) is a computational method that determines whether an a priori defined set of genes shows statistically significant, concordant differences between two biological states (e.g., phenotypes). This research tested whether the autoimmune discovery panel (priori set of genes) showed differences between ulcerative colitis versus healthy controls (43 cases, 13 controls, and 30 ulcerative colitis). The GSEA showed enrichment of the pathway toward the ulcerative colitis patients. The most relevant genes of the leading edge were *IL1RN*, *MMP3*, *OSMR*, *FCGR3B*, *FCGR3A*, *TNC*, *TNFRSF6b*, *CD274* (*PD-L1*), *PLAU*, and *S100A9*. In Figure 2, the GSEA plot is shown.

### 3.3. Machine Learning and Artificial Neural Networks

#### 3.3.1. Ulcerative Colitis Versus Healthy Controls

**Figure 3:**
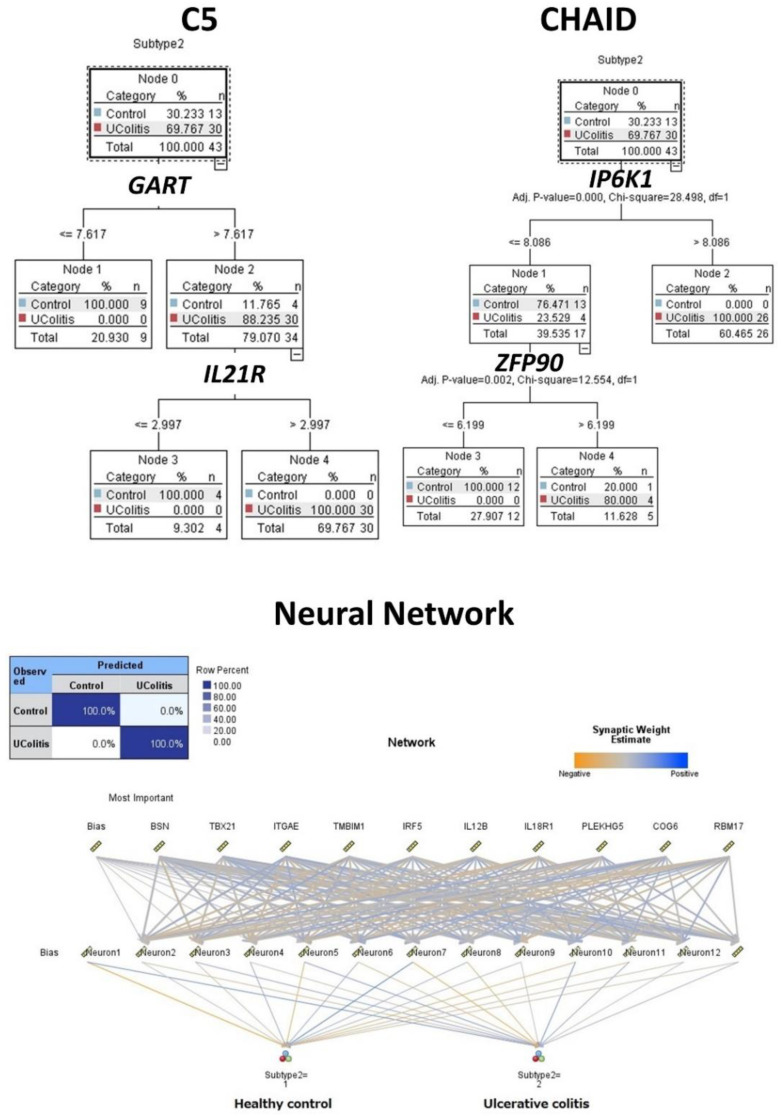
Modeling ulcerative colitis versus healthy controls using C5 tree, CHAID tree, and artificial neural networks. Several machine learning techniques, including artificial neural networks, were used to predict ulcerative colitis using gene expression data from the autoimmune discovery panel. This figure shows the results of the C5 tree (which used *GART* and *IL21R* genes in the final model), CHAID tree (*IP6K1* and *ZFP90*), and the neural network (which used the 734 genes of the autoimmune discovery panel). The accuracy of these 3 methods was high, 100%, 98%, and 100%, respectively.

**Figure 4:**
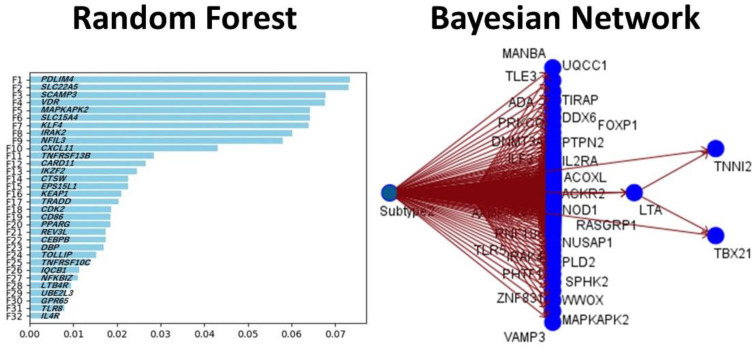
Modeling ulcerative colitis versus healthy controls using random forest and Bayesian network. This figure shows the results of the modeling of the prediction of ulcerative colitis against healthy controls using gene expression data of the autoimmune discovery panel. The random forest plot shows the genes of the model, ranked according to their predicted importance. The Bayesian network also predicted the ulcerative colitis cases (subtype 2 in the figure). The Bayesian network shows the genes (nodes) and the probabilistic, or conditional, independencies between them. The causal relationships may be represented, but the links (arcs) of the network do not necessarily represent direct cause and effect.

The same gene expression matrix with the autoimmune discovery panel of the GSEA analysis was used to predict ulcerative colitis status (*n* = 30) against healthy controls (*n* = 13). A total of 16 models were used, including C5, logistic regression, Bayesian network, discriminant analysis, KNN algorithm, LSVM, random trees, SVM, Tree-AS, XGBoost linear, XGBoost tree, CHAID, Quest, C&R tree, random forest, and neural network. Table 1 lists the models in order according to the overall accuracy, and the number of genes used in each final model are also shown. Figure 3 and Figure 4 show some of the predictive and classification models. 

#### 3.3.2. Ulcerative Colitis (Involved Active, Non-Involved Active, and Inactive/Remission) Versus Healthy Controls

**Figure 5:**
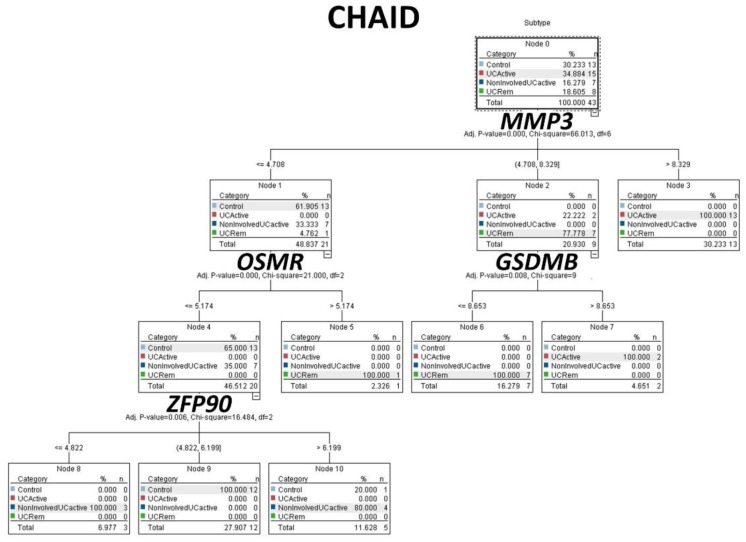
Modeling ulcerative colitis versus healthy controls. The target variable was the disease, ulcerative colitis (involved active (2), non-involved active (3), and inactive/remission (4)), and healthy controls (1). Using a CHAID tree and the gene expression of 4 genes (*MMP3*, *OSMR*, *GSDMB*, and *ZFP90*) it was possible to classify for histological subtypes with 97.7% accuracy.

**Figure 6:**
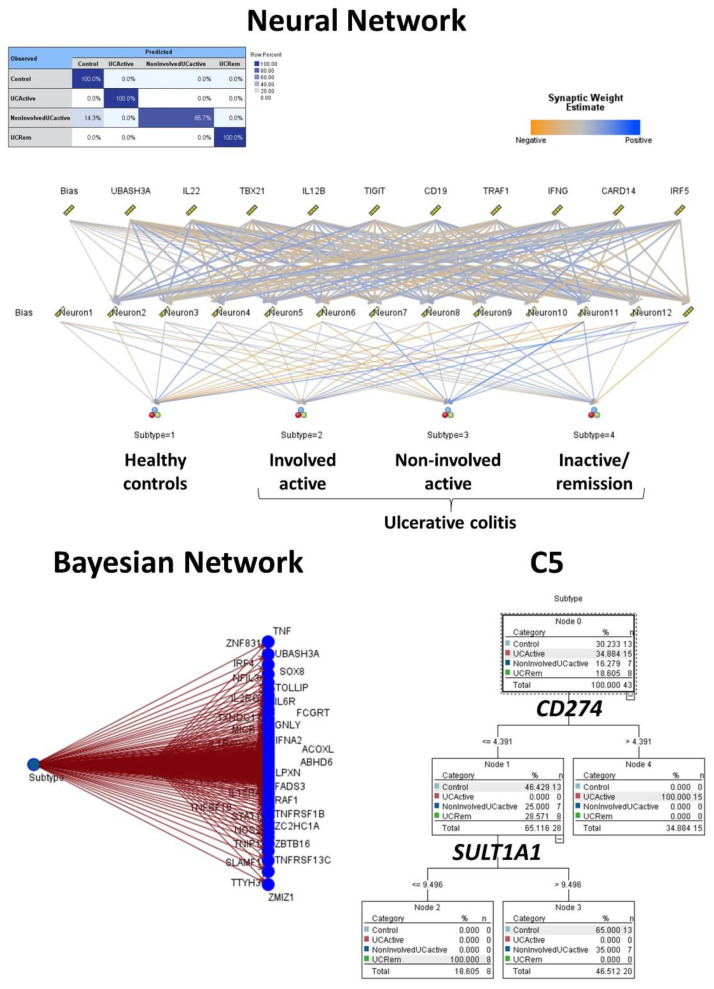
Modeling ulcerative colitis versus healthy controls. The target variable was the disease, ulcerative colitis (involved active (2), non-involved active (3), and inactive/remission (4)), and healthy controls (1). Using an artificial neural network, it was possible to classify the patients with 97.7% accuracy; the most relevant gene for predicting the subtype was *UBASH3A*. The modeling was also complete with a Bayesian network and C5 tree. Of note, C5 tree only used 2 genes, the *CD274* (*PD-L1*) and *SULTA1*, and had an accuracy of 83.7%.

The same procedure was repeated, including the same gene expression matrix with the autoimmune discovery panel as predictors. The series comprised a total number of 43 biopsies, including 13 healthy controls, 8 inactive ulcerative colitis, 7 non-involved active ulcerative colitis, and 15 involved active ulcerative colitis. The target variable was the disease, ulcerative colitis (involved active (coded as number/output “2”), non-involved active (“3”), and inactive/remission (“4”)), and healthy controls (“1”). Several analyses were performed, and the results of the overall accuracy (%) and the number of genes (fields) used are shown in Table 2, Figure 5 and Figure 6. 

### 3.4. Validation of CD274 (PD-L1) in an Independent Series

**Figure 7:**
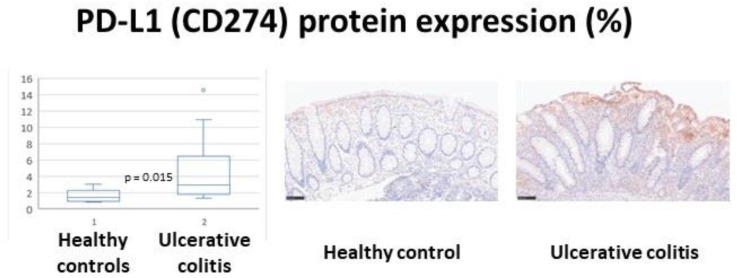
Programmed cell death factor 1 (*PD-L1*, *CD274*) expression in ulcerative colitis. Ulcerative colitis is characterized by increased *PD-L1* expression more than healthy controls (*p* = 0.015). Ulcerative colitis samples were characterized by disruption of the epithelial layer, inflammation of the lamina propria, crypt branching, shortening, and disarray.

Programmed cell death factor 1 (*PD-L1*, *CD274*) was a marker identified both in the conventional gene expression analysis using the GEO2R and GSEA, and in the machine learning analyses (Table 2). Twenty cases from a recent publication that included five healthy controls and fifteen with ulcerative colitis. The primary antibody was that used was the *PD-L1* (extracellular domain-specific) (E1J2J) Rabbit mAb #15165 (CST). The slides were evaluated under the optical microscope and *PD-L1* quantified using Fiji software (NIH). The bioinformatics analysis was confirmed and showed that ulcerative colitis is characterized by increased *PD-L1* protein expression. Therefore, the *CD274* (*PD-L1*) marker was validated in another series of cases. Ulcerative colitis versus healthy controls (mean ± STD): 4.7% ± 3.8 versus 1.6% ± 0.9 (*p* = 0.015) (Figure 7). The cases were endoscopic biopsies, selected from Japanese patients from 2005 to 2013. The selection criteria were biopsies taken in the colonoscopy at diagnosis, and the presence of adequate tissue for histological evaluation. When multiple biopsies were present, the most inflamed was chosen [7]. The clinicopathological characteristics of these 20 cases are shown in Appendix A Table A1, which includes the Geboes histologic disease activity and the Baron endoscopic scores. The digital images are shown as Supplementary Data and uploaded to Zenodo platform as a zip file (Carreras, Joaquim. (2022). healthcare-10-01476 (Version 1). Zenodo. https://doi.org/10.5281/zenodo.6956123) (accessed on 3 August 2022).

## 4. Discussion

Machine learning is a branch of artificial intelligence (AI) that uses data and algorithms similarly to humans, improving its accuracy progressively. Machine learning has become an important field in data science. By using several statistical techniques, predictions and classifications are made through trained algorithms. Data mining projects use machine learning techniques to understand the underlying mechanisms. Eventually, these insights drive decision making within many types of applications, including in the medical field. Since big data in medicine is continuously expanding, the necessity of advanced data analysis is crucial. Machine learning algorithms are usually created using frameworks, such as TensorFlow, Keras, and Pytorch, that accelerate solution development [19,20,21].

The term artificial intelligence includes several subfields as machine learning, deep learning, and neural networks. Nonetheless, neural networks are a sub-discipline of machine learning, and deep learning is a sub-discipline of neural networks. The difference between deep learning and machine learning depends on how the algorithm learns. Classical “not-deep” machine learning requires more structured data to learn, and the human intervention, in the form of human determination of features to analyze and understand the differences between data inputs. However, “deep” machine learning can use labeled datasets (known as supervised learning) and also use raw unstructured data, and they can automatically determine features that differentiate categories of data, enabling the use of large datasets [19,20,21]. 

The basic structure of an artificial neural network is composed of an input layer, one or more hidden layers, and an output layer. These layers contain nodes (neurons). Each node connects to another and has an associated weight and threshold. When the output of an individual node is above the specified threshold, the node is activated and sends data to the next network layer. Contrarily, an output below the threshold does not send data to the next layer. The term “deep” refers to the number of layers of the network. More than three layers (including the input and output layers) is considered a deep learning algorithm. A basic neural network would only have three layers [19,20,21].

There are several commonly used machine learning algorithms, including neural networks, liner regression, logistic regression, clustering, decision trees, and random forests. This research used several machine learning techniques, including C5, logistic regression, Bayesian network, discriminant analysis, KNN algorithm, LSVM, random trees, SVM, Tree-AS, XGBoost linear, XGBoost tree, CHAID, Quest, C&R tree, random forest, and neural network. The predictors were 755 gens of an autoimmune discovery panel that contained genes closely associated with germline variants across nine different autoimmune diseases or relevant to the immune response. The autoimmune disease coverage included multiple sclerosis, rheumatoid arthritis, systemic lupus erythematosus, type 1 diabetes, ankylosing spondylitis, celiac disease, ulcerative colitis, inflammatory bowel disease, and psoriasis. The target variable was the distinction between ulcerative colitis versus healthy donors, or the three variants of ulcerative colitis (involved mucosa active, non-involved mucosa of active, and inactive/remission) versus healthy donors. Each machine learning method provided a final model with a different overall accuracy using a defined set of genes of the panel. This research did not just compare the different models but provided different solutions to predict ulcerative colitis and to try understanding the pathogenesis. Of note, low accuracy solutions are to be discarded.

Detailed descriptions of the clinicopathological features of ulcerative colitis have been recently published [22,23,24,25,26,27,28,29,30]. A gene that was highlighted in this research was *CD274* (*PD-L1*). This marker belongs to the immune checkpoint, and it is important for inhibiting the host immune response. By immunohistochemistry, it was confirmed that high expression of *PD-L1* was characteristic of ulcerative colitis. We recently described the role of *PD-L1* in a DSS colitis model [31]. Other genes that were highlighted were *FCGR3A*, *GSDMB*, *IFNG*, *IRF5*, *MMP3*, *OSMR*, *SULT1A1*, *TGFBI*, and *ZFP90* (among others). These genes belong to the ulcerative colitis autoimmune coverage of the discovery panel, but also belong to Crohn’s disease, celiac disease, and other immune response genes. Therefore, these markers are expected to be relevant not only to ulcerative colitis but also to other autoimmune diseases. For example, polymorphisms of *FCGR3A* are associated with susceptibility to ulcerative colitis [32]; gene expression genotype analysis identified *GSDMB* as a contributor to inflammatory bowel disease susceptibility [33]; distinct *IFNG* methylation status was found in a subset of ulcerative colitis patients based on reactivity to microbial antigens [34], reducing *IRF5* expression attenuated colitis in mice but impairing the clearance of intestinal pathogens [35]; and in children, *MMP3* was correlated with several clinical and endoscopic activity on ulcerative colitis in children [36].

This study used a series of 43 cases of gene expression to identify ulcerative colitis markers, and the *PD-L1* marker was validated in an independent series of 20 cases. The number of cases is a limitation. Artificial intelligence tools, especially neural networks, are very powerful techniques for deciphering patterns even using a small series of cases, but the results of this research will have to be validated in a larger series of cases.

In conclusion, using an autoimmune discovery gene expression panel and several machine learning techniques, it was proved that it is possible to predict ulcerative colitis and identify pathogenic markers.

## Figures and Tables

**Table 1:** Prediction of ulcerative colitis using machine learning and artificial neural network modeling.

Model	Overall Accuracy (%)	No. Fields (Genes) Used	Most Relevant Genes
C5	100	2	*GART*, *IL21R*
Logistic regression	100	734	*AAMP*, *ABHD6*, *ACKR2*, *ACOXL*, *ACSL6*, *ADA*, *ADAM30*, *ADCY3*, *ADCY7*, *AFF3*, *AGAP2*, *AHI1*, *AHR*, *AIRE*, *ANKRD55*, *ANTXR2*, *APEH*, *APOBEC3G*, *ARG1*, *ARHGAP30*, *ARID5B*, *ARPC2*, *ATF4*, *ATG16L1*, *ATG5*, *ATM*, *B2M*, *B3GNT2*, *BABAM2*, *BACH2*, *BAD*, *BANK1*, *BATF*, *BATF3*, *BCL10*, *BCL3*, *BCL6*, *BID*, *BLK*, *BLNK*, *BORCS5*, and *BSN.*
Discriminant	100	734	-
LSVM	100	734	*CCL11*, *IL1RN*, *MMP3*, *CXCL3*, *FCGR3A*, *TLR3*, *NFIL3*, *TTYH3*, *NLRP2*, and *OSMR*
SVM	100	734	-
XGBoost Linear	100	734	-
XGBoost Tree	100	734	-
Neural Network	100	734	*BSN*, *TBX21*, *ITGAE*, *TMBIM1*, *IRF5*, *IL12B*, *IL18R1*, *PLEKHG5*, *COG6*, and *RBM17*
CHAID	97.7	2	*IP6K1*, *ZFP90*
Random Forest	97.7	734	*PDLIM4*, *SLC22A5*, *SCAMP3*, *VDR*, *MAPKAPK2*, *SLC15A4*, *KLF4*, *IRAK2*, *NFIL3*, and *CXCL11*
KNN Algorithm	95.4	734	-
C&R Tree	95.4	12	*METTL1*, *ADA*
Quest	83.7	6	*IRAK1*
Bayesian Network	65.1	734	-
Random Trees	0	734	N/A

**Table 2:** Prediction of ulcerative colitis (active, non-involved active, and inactive) using machine learning and artificial neural network modeling.

Model	Overall Accuracy (%)	No. Fields (Genes) Used	Most Relevant Genes
Logistic regression	100	734	-
Discriminant	100	734	-
SVM	100	734	-
XGBoost Linear	100	734	-
XGBoost Tree	100	734	-
CHAID	97.7	4	*MMP3*, *OSMR*, *ZFP90*, and *GSDMB*
Random Forest	97.7	734	*TLR2*, *IFNAR2*, *BID*, *NCF2*, *IDO1*, *FCGR1A*, *CSF2RB*, *TGFBI*, *S1PR1*, and *IRAK1*
Neural Network	97.7	734	*UBASH3A*, *IL22*, *TBX21*, *IL12B*, *TIGIT*, *CD19*, *TRAF1*, *IFNG*, *CARD14*, and *IRF5*
Bayesian Network	95.4	734	-
KNN Algorithm	93.0	734	-
LSVM	86.1	734	-
C5	83.7	2	*CD274* and *SULT1A1*
C&R Tree	65.1	6	*CD274*
Quest	62.8	6	*FCGR3A*
Random Trees	0	734	N/A

## Data Availability

All the data, including methodology, are available upon reasonable request to Dr. Joaquim Carreras (joaquim.carreras@tokai-u.jp). The histological images of *PD-L1* can be accessed at https://doi.org/10.5281/zenodo.6956123 (accessed on 3 August 2022). List of genes: https://doi.org/10.5281/zenodo.6957666 (accessed on 3 August 2022).

## Appendix A

**Table A1:** Clinicopathological characteristics of the cases of ulcerative colitis.

Type	*PD-L1* (%)	Biopsy	Age	Sex	Baron Score	Geboes Score
Control	0.84	Rectum	64	Male	-	-
Control	1.14	Descending	56	Male	-	-
Control	1.45	Descending	59	Male	-	-
Control	1.45	Rectum	26	Male	-	-
Control	3.09	Rectum	59	Female	-	-
Ulcerative colitis	1.38	Rectum	51	Male	1	1
Ulcerative colitis	1.47	Sigmoid	31	Female	2	2
Ulcerative colitis	1.61	Rectum	37	Female	1	2
Ulcerative colitis	1.80	Rectum	37	Female	2	2
Ulcerative colitis	2.06	Rectum	33	Male	2	3
Ulcerative colitis	2.24	Rectum	77	Female	2	2
Ulcerative colitis	2.97	Rectum	46	Male	1	3
Ulcerative colitis	2.98	Sigmoid	41	Male	2	3
Ulcerative colitis	4.74	Rectum	59	Male	1	2
Ulcerative colitis	6.34	Rectum	23	Male	2	2
Ulcerative colitis	4.04	Rectum	22	Female	2	4
Ulcerative colitis	6.52	Sigmoid	43	Female	3	2
Ulcerative colitis	6.89	Descending	54	Female	2	4
Ulcerative colitis	10.99	Rectum	20	Male	3	4
Ulcerative colitis	14.55	Descending	17	Female	2	2
